# An evaluation in vitro of PARP-1 inhibitors, rucaparib and olaparib, as radiosensitisers for the treatment of neuroblastoma

**DOI:** 10.1186/s12885-016-2656-8

**Published:** 2016-08-11

**Authors:** Donna L. Nile, Colin Rae, Iain J. Hyndman, Mark N. Gaze, Robert J. Mairs

**Affiliations:** 1 Radiation Oncology, Institute of Cancer Sciences, University of Glasgow, Glasgow, UK; 2 University College London Hospitals, London, UK

**Keywords:** Neuroblastoma, ^131^I-MIBG, Targeted radiotherapy, PARP-1, DNA damage

## Abstract

**Background:**

The radiopharmaceutical ^131^I-meta-iodobenzylguanidine (^131^I-MIBG) is an effective treatment for neuroblastoma. However, maximal therapeutic benefit from ^131^I-MIBG is likely to be obtained by its combination with chemotherapy. We previously reported enhanced antitumour efficacy of ^131^I-MIBG by inhibition of the poly(ADP-ribose) polymerase-1 (PARP-1) DNA repair pathway using the phenanthridinone derivative PJ34. Recently developed alternative PARP-1 inhibitors have greater target specificity and are expected to be associated with reduced toxicity to normal tissue. Therefore, our purpose was to determine whether the more specific PARP-1 inhibitors rucaparib and olaparib enhanced the efficacy of X-radiation or ^131^I-MIBG.

**Methods:**

Radiosensitisation of SK-N-BE(2c) neuroblastoma cells or noradrenaline transporter gene-transfected glioma cells (UVW/NAT) was investigated using clonogenic assay. Propidium iodide staining and flow cytometry was used to analyse cell cycle progression. DNA damage was quantified by the phosphorylation of H2AX (γH2AX).

**Results:**

By combining PARP-1 inhibition with radiation treatment, it was possible to reduce the X-radiation dose or ^131^I-MIBG activity concentration required to achieve 50 % cell kill by approximately 50 %. Rucaparib and olaparib were equally effective inhibitors of PARP-1 activity. X-radiation-induced DNA damage was significantly increased 2 h after irradiation by combination with PARP-1 inhibitors (10-fold greater DNA damage compared to untreated controls; *p* < 0.01). Moreover, combination treatment (i) prevented the restitution of DNA, exemplified by the persistence of 3-fold greater DNA damage after 24 h, compared to untreated controls (*p* < 0.01) and (ii) induced greater G_2_/M arrest (*p* < 0.05) than either single agent alone.

**Conclusion:**

Rucaparib and olaparib sensitise cancer cells to X-radiation or ^131^I-MIBG treatment. It is likely that the mechanism of radiosensitisation entails the accumulation of unrepaired radiation-induced DNA damage. Our findings suggest that the administration of PARP-1 inhibitors and ^131^I-MIBG to high risk neuroblastoma patients may be beneficial.

## Background

Despite an incidence rate of 6 % of all childhood cancers [1], neuroblastoma is responsible for 15 % of all childhood cancer deaths [2]. Tumours originate from tissues derived from primordial neural crest cells and subsequently can arise anywhere in the sympathetic nervous system [3]. Fifty percent of all primary tumours manifest in the adrenal medulla [2]. Patients with high risk disease undergo multimodal treatment, involving intensive chemo- and radiotherapy following surgical resection. However, despite rigorous treatment, there is only a 40 % overall survival rate [2]. This could possibly be improved with immunotherapy, which has proven an effective treatment for high-risk neuroblastoma patients in remission [4], but further improvements are necessary to limit adverse cytotoxic effects.

Ninety percent of neuroblastoma tumours express the noradrenaline transporter (NAT) [5], allowing the active uptake of catecholamine neurotransmitters. Targeted radiotherapy using radioiodinated meta-iodobenzylguanidine (^131^I-MIBG) exploits this characteristic of neuroblastoma cells. The radiopharmaceutical ^131^I-MIBG is a structural analogue of noradrenaline, facilitating its selective accumulation by neuroblastoma tumour cells. ^131^I-MIBG has demonstrated efficacy as a single agent [6, 7]. However, the optimal use of ^131^I-MIBG has yet to be defined [8], and increasingly it is administered in combination with cytotoxic drug therapy [9–11]. Indeed, a Clinical Oncology Group pilot study (NCT01175356/ANBL09P1) is currently investigating the efficacy of ^131^I-MIBG in combination with intensive induction chemotherapy in high-risk neuroblastoma patients.

Poly(ADP-ribose) polymerases (PARPs) mediate the post-translational modification of target proteins following hydrolysis of the PARP substrate, nicotinamide adenine dinucleotide (NAD^+^) [12, 13]. The first discovered PARP enzyme, and hence the most comprehensively studied, is PARP-1 [14, 15]. Upon detection of DNA strand breaks, PARP-1 catalytic activity is increased 500-fold [13], resulting in the ADP-ribosylation of target proteins including histones, components of DNA repair pathways and PARP-1 auto-modification [16]. PARP-1 inhibition was shown to exhibit synthetic lethality in cells lacking *BRCA-1* and *BRCA-2* [17, 18], two important components of homologous recombination repair of DNA double strand breaks [19]. Inhibition of PARP-1 function in BRCA-deficient cell lines, either by genetic silencing of *PARP-1* [18] or pharmacologically using a PARP-1 inhibitor [17], prompted the accumulation of DNA lesions that were not repaired by homologous recombination.

PARP-1 inhibitors have shown great promise when used in combination with treatments that cause substantial DNA damage, including ionising radiation [20–23], DNA alkylating agents [20, 24] and the topoisomerase-1 poisons topotecan or irinotecan [25, 26]. Indeed, we have shown previously that the second generation PARP-1 inhibitor PJ34 enhanced the efficacy of 3-way modality treatment involving ^131^I-MIBG and topotecan [22]. However, it has been suggested that PJ34 may be toxic to normal cells [27, 28]. Innovative PARP-1 inhibitors, such as olaparib and rucaparib, have greater specificity, enhanced target affinity, and have now progressed to clinical evaluation [12, 16, 29]. Rucaparib was the first PARP-1 inhibitor to enter clinical trials [30] and olaparib was the first PARP-1 inhibitor to gain FDA approval for the treatment of germline *BRCA*-deficient ovarian cancer. Both rucaparib and olaparib have shown promise in phase II/III clinical trials, both as monotherapies in *BRCA*-mutated breast cancer [31], ovarian cancer [32] and prostate cancer [33], and in combination with cytotoxic drug therapy [34–36].

Therefore, PARP-1 inhibition is a promising approach not only to the targeting of BRCA-deficient cancers which are deficient in DNA repair capacity, but also to the enhancement of the efficacy of DNA damaging chemo- and radiotherapies. Indeed, increased PARP-1 expression has previously been associated with greater neuroblastoma cell genomic instability, higher neuroblastoma stage and poor overall survival [37], suggesting these tumours will be susceptible to PARP-1 inhibition. PARP-1 inhibitors are also being evaluated clinically for the treatment of children with refractory or recurrent malignancies, such as solid neoplasms, acute lymphoblastic leukaemia, central nervous system neoplasms and neuroectodermal tumours (NCT02116777/ADVL1411). In the present study, we determined the radiosensitising potential of rucaparib and olaparib, two PARP-1 inhibitors currently undergoing phase II/III clinical investigation, in combination with external beam X-radiation or the neuroblastoma-targeting radiopharmaceutical ^131^I-MIBG. We also examined the effect of combination treatment on cell cycle progression and the persistence of DNA damage.

## Methods

### Reagents

Rucaparib and olaparib were purchased from Selleckchem (Suffolk, UK) and were reconstituted using phosphate buffered saline (PBS) and dimethyl sulfoxide (DMSO), respectively. Drugs were then diluted in culture medium, maximum DMSO concentration was 0.2 % (v/v). Unless otherwise stated, all other cell culture reagents were purchased from Life Technologies (Paisley, UK) and all chemicals were purchased from Sigma-Aldrich (Poole, UK).

### Cell culture

Human neuroblastoma SK-N-BE(2c) cells were purchased from the American Type Culture Collection. SK-N-BE(2c) cells were maintained in high glucose Dulbecco’s Modified Eagle Medium (DMEM) containing 15 % (v/v) foetal calf serum, 2 mM L-glutamine and 1 % (v/v) non-essential amino acids. Human glioblastoma UVW cells [38] were transfected with a plasmid containing the bovine noradrenaline transporter (NAT) gene [39]. UVW/NAT cells were maintained in Minimum Essential Medium (MEM) containing 10 % (v/v) foetal calf serum, 2 mM L-glutamine, 1 % (v/v) non-essential amino acids and 1 mg/ml geneticin. Cells were incubated at 37 °C, 5 % CO_2_ in a humidified incubator, and were passaged every 3-4 days. Cell lines were cultured in this study for less than 6 months after resuscitation.

### Clonogenic assay

Monolayers were cultured at a density of 10^5^ cells in 25 cm^2^ flasks. Cells in the exponential growth phase were treated with fresh culture medium containing rucaparib or olaparib and were simultaneously irradiated using an Xstrahl RS225 X-Ray irradiator (Xstrahl Limited, Surrey, UK) at a dose rate of 0.93 Gy/min. After 24 h incubation at 37 °C, cells were seeded in 21.5 cm^2^ petri dishes at a density of 500 (SK-N-BE(2c)) or 250 (UVW/NAT) cells per dish in triplicate. After 8 days (UVW/NAT) or 14 days (SK-N-BE(2c)), colonies containing ≥50 cells were fixed with 50 % (v/v) methanol in PBS and stained with crystal violet. Stained colonies were counted and expressed as a fraction of the untreated, unirradiated control. Radiation survival curves were fitted assuming a linear-quadratic relationship between survival and radiation dose using GraphPad Prism 5.01 (GraphPad Software, San Diego, USA). The data were used to calculate the dose required to sterilise 50 % of clonogens (IC_50_), as well as the dose-enhancement factor at IC_50_ (DEF_50_).

### PARP-1 activity assay

Cells were seeded at a density of 1x10^5^ (SK-N-BE(2c)) or 0.5x10^5^ (UVW/NAT) cells on to glass coverslips in 6-well plates. After 48 h, fresh medium was added containing rucaparib or olaparib, before incubating for 1.5 h at 37 °C. PARP-1 activity was stimulated by treatment with 20 mM hydrogen peroxide for 20 min at room temperature in the dark. PBS or DMSO treatment of 0.09 % (v/v) in medium constituted negative controls. Cells were fixed with ice cold methanol/acetone (1:1) on ice for 15 min, before blocking with 2 % (w/v) bovine serum albumin (BSA) in PBS for 30 min at room temperature. Fixed cells were incubated for 1 h at room temperature with a 1:200 dilution of mouse anti-PADPR monoclonal antibody (Abcam, Cambridge, UK; Cat# ab14459) in antibody buffer (10 mM Tris–HCl pH 7.5, 150 mM NaCl, 0.1 % (w/v) BSA in distilled water). Bound anti-PADPR primary antibody was visualised after 1 h incubation at room temperature using goat anti-mouse Alexa Fluor 488-conjugated secondary antibody (Life Technologies, Paisley, UK; Cat# A11029), at a dilution of 1:500 in antibody buffer. Cells were fixed by treatment with 4 % (w/v) paraformaldehyde for 30 min at room temperature in the dark, before mounting on to microscope slides using Vectashield mounting medium containing DAPI nuclear stain (Vector Laboratories, Peterborough, UK). Fluorescence was visualised by means of a Zeiss Axio Observer LSM 780 confocal microscope, using identical laser power and gain settings for all images.

### ^131^I-MIBG synthesis and treatment

No-carrier-added (n.c.a.) ^131^I-MIBG was prepared using a solid phase system wherein the precursor of ^131^I-MIBG was attached to an insoluble polymer via the tin-aryl bond [40, 41]. The reaction conditions, HPLC purification procedure, and radiochemical yield were as described previously [42]. Cells were treated with ^131^I-MIBG for 2 h, by which time ^131^I-MIBG uptake was maximal [43].

### Fluorescence Activated Cell Sorting (FACS) analysis

Cells were seeded at a density of 7x10^5^ (SK-N-BE(2c)) or 4x10^5^ (UVW/NAT) cells into 75 cm^2^ flasks. After 48 h, fresh medium was added containing rucaparib or olaparib and cells were simultaneously irradiated before incubating for 1.5 h at 37 °C. Cells were trypsinised and washed with PBS, before fixing with 70 % (v/v) ethanol in water at -20 °C. Ethanol was removed by washing with PBS. Cells were permeabilised by treatment with 0.05 % (v/v) Triton X-100 in PBS containing a 1:50 dilution of rabbit anti-phospho-Histone H2AX(Ser139)-Alexa Fluor 647-conjugated monoclonal antibody. After 40 min incubation at room temperature, excess antibody was removed by washing with PBST buffer (0.1 % (v/v) Tween 20 in PBS). Finally, cell pellets were resuspended in PBS containing propidium iodide (10 μg/ml) and RNase A (200 μg/ml), before analysis using a BD FACSVerse flow cytometer (BD BioSciences, Oxford, UK). FACS data were quantified using FlowJo 7.6.5 software. For cell cycle analysis, cells were treated separately, and were incubated with propidium iodide and RNase A only as detailed above.

### γH2AX immunofluorescent microscopy

Cells were seeded as for PARP-1 activity assay. Fresh medium was added containing rucaparib or olaparib, and cells were simultaneously irradiated, before incubating for 1.5 h at 37 °C. After treatment, cells were fixed with 4 % (w/v) paraformaldehyde for 30 min at room temperature before blocking with 2 % (w/v) BSA (in PBS) for 30 min at room temperature. Fixed cells were then incubated overnight at 4 °C with a 1:50 dilution of rabbit anti-phospho-Histone H2AX(Ser139)-Alexa Fluor 647-conjugated monoclonal antibody (Cell Signalling Technology, supplied by New England Biolabs, Hitchin, UK, Cat# 9720), followed by overnight incubation with a 1:250 dilution of mouse anti-β-tubulin (Life Technologies, Paisley, UK) in antibody buffer (10 mM Tris–HCl pH 7.5, 150 mM NaCl, 0.1 % (w/v) BSA in distilled water). Bound anti-β-tubulin primary antibody was visualised after 1 h incubation at room temperature using goat anti-mouse Alexa Fluor 488-conjugated secondary antibody (Life Technologies, Paisley, UK; Cat# A11029), at a dilution of 1:500 in antibody buffer. Cells were mounted and fluorescence visualised as for PARP-1 activity assay.

### Statistical analysis

All statistical analyses were performed using GraphPad Prism version 5.01 (GraphPad Software, California, USA). The number of experimental repeats is provided in figure legends. Data are presented as means ± standard error of the mean (SEM). Statistical significance was determined by either the unpaired Student’s two-tailed *t* test, or the one-way ANOVA followed by post-hoc testing using Bonferroni correction for multiple comparisons. A probability (*p*) value < 0.05 was considered statistically significant and < 0.01 highly significant.

## Results

### Rucaparib and olaparib at concentrations ≤ 1 μM are not cytotoxic

**Fig. 1:**
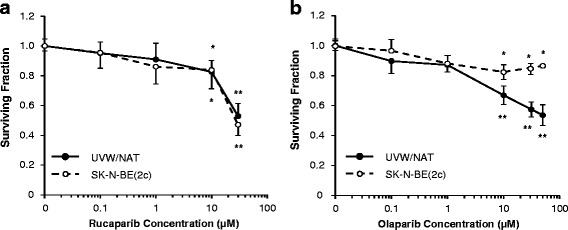
The effect of rucaparib and olaparib on clonogenic survival. SK-N-BE(2c) (**a**) and UVW/NAT cells (**b**) were treated with various concentrations of rucaparib or olaparib. After 24 h treatment, clonogenic survival was assessed by clonogenic assay. Data are means ± SEM, *n* = 3; * *p* < 0.05, ** *p* < 0.01 compared to untreated control

Neither rucaparib nor olaparib was cytotoxic at 1 μM. Minor yet significant clonogenic cell kill was induced by both drugs at 10 μM. Rucaparib at 30 μM was significantly toxic to SK-N-BE(2c) and UVW/NAT cells (Fig. 1a; *p* < 0.01). Neither cell line survived 24 h treatment with 50 μM rucaparib. In contrast, 30 μM olaparib, though highly toxic to UVW/NAT cells (*p* < 0.01), induced modest kill of SK-N-BE(2c) clonogens (Fig. 1b; *p* < 0.5).

### Rucaparib and olaparib inhibited PARP-1 activity in SK-N-BE(2c) and UVW/NAT cells

**Fig. 2:**
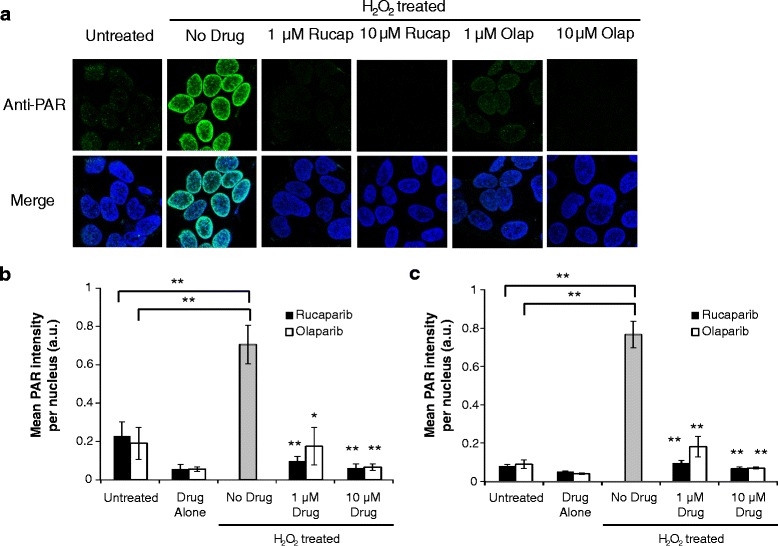
The effect of rucaparib and olaparib on PARP-1 activity. Cells were treated with 20 mM hydrogen peroxide (H_2_O_2_) in order to stimulate PARP-1 activity in the presence or absence of the PARP-1 inhibitors rucaparib and olaparib. Poly(ADP-ribose) (PAR) chain synthesis was detected using an anti-PAR monoclonal Alexa Fluor 488-conjugated antibody (green). The nucleus was visualised using the nuclear counterstain DAPI (blue). Representative images obtained from the analysis of anti-PAR staining of SK-N-BE(2c) cells are shown (**a**). Fluorescence intensity for Alexa Fluor 488 was quantified using ImageJ software and normalised to DAPI fluorescence intensity in SK-N-BE(2c) (**b**) and UVW/NAT (**c**) cells. Drug vehicles were PBS and DMSO for rucaparib and olaparib, respectively. Untreated cells were exposed to 0.09 % (v/v) drug vehicle diluted in culture medium. The designation ‘Drug Alone’ indicates that cells were treated with 10 μM PARP-1 inhibitor in the absence of H_2_O_2_. The designation’No Drug’ indicates that cells were treated with 20 mM H_2_O_2_ alone, in the absence of PARP-1 inhibitor. Data are means ± SEM, *n* = 3; *p < 0.05, ** *p* < 0.01 compared to H_2_O_2_ alone

Incubation with 10 μM rucaparib or olaparib (termed drug alone in Fig. 2) induced a 50 % reduction in endogenous PARP-1 activity compared with cells which were treated only with the drug vehicle. In contrast, PARP-1 activity was significantly enhanced by treatment with the DNA damaging agent hydrogen peroxide (H_2_O_2_) at 20 mM – labelled no drug on Fig. 2. This was demonstrated by a 3.5-fold (*p* < 0.01) and 9.4-fold (*p* < 0.01) increase in PARP-1 activity compared to untreated SK-N-BE(2c) (Fig. 2b) and UVW/NAT cells (Fig. 2c), respectively. However, the H_2_O_2_-induced increase in PARP-1 activity was reduced to levels comparable with untreated cells after treatment with 1 μM or 10 μM rucaparib or olaparib in both cell lines (*p* < 0.01).

### PARP-1 inhibition sensitised cells to X-radiation and ^131^I-MIBG treatment

To investigate the radiosensitising potential of rucaparib and olaparib in SK-N-BE(2c) and UVW/NAT cells, clonogenic survival was assessed following drug treatment in simultaneous combination with external beam X-irradiation or treatment with the NAT-targeting radiopharmaceutical ^131^I-MIBG. In combination treatments, PARP-1 inhibitors were administered at non-cytotoxic (1 μM) or cytotoxic (10 μM and 30 μM) concentrations, all of which inhibited PARP-1 activity in both cell lines.

**Fig. 3:**
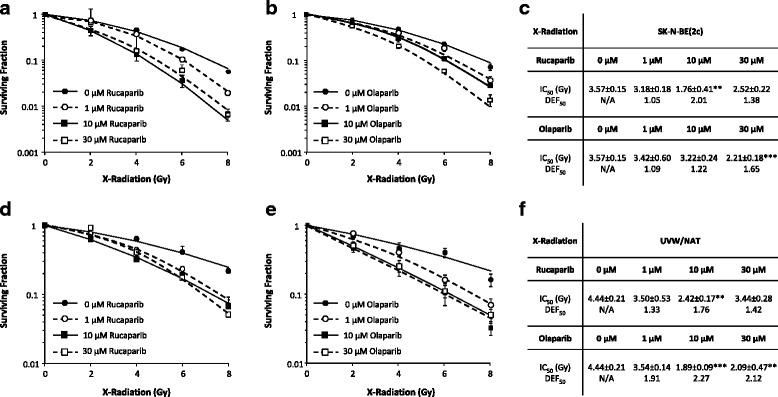
The effect of PARP-1 inhibition in combination with external beam X-radiation. SK-N-BE(2c) (**a**, **b**, **c**) and UVW/NAT cells (**d**, **e**, **f**) were treated simultaneously with rucaparib (**a**, **d**) or olaparib (**d**, **e**) in combination with a range of doses of external beam X-radiation. Cells were treated with rucaparib or olaparib, at concentrations of 1 μM, 10 μM or 30 μM, following exposure to a range of doses of X-radiation, for 24 h prior to seeding cells for clonogenic assay. The X-irradiation dose associated with 50 % cell kill (IC_50_) and the dose enhancement factors corresponding to 50 % clonogenic cell kill (DEF_50_) are presented for SK-N-BE(2c) (**c**) and UVW/NAT (**f**) cells. Data are means ± SEM, *n* = 3; **p < 0.01, *** *p* < 0.001 compared to the IC_50_ in the absence of drug

Rucaparib and olaparib sensitised SK-N-BE(2c) cells (Fig. 3a and b) and UVW/NAT cells (Fig. 3d and Fig. 3e) to X-irradiation. This was indicated by the reduced X-radiation dose required to achieve 50 % cell kill (IC_50_). In the absence of PARP-1 inhibition, the IC_50_ value corresponding to X-radiation treatment alone of SK-N-BE(2c) cells was 3.57 ± 0.15 Gy (Fig. 3c). This value was decreased to 3.18 ± 0.18, 1.76 ± 0.41 (*p* < 0.01) or 2.52 ± 0.22 Gy by treatment with 1, 10 or 30 μM rucaparib, respectively. Exposure to 1, 10 or 30 μM olaparib reduced IC_50_ values to 3.42 ± 0.60, 3.22 ± 0.24 and 2.21 ± 0.18 Gy (*p* < 0.001) respectively, in SK-N-BE(2c) cells. Likewise in UVW/NAT cells, IC_50_ values observed after exposure to X-radiation alone, or in the presence of 1, 10 or 30 μM rucaparib were 4.44 ± 0.21, 3.50 ± 0.53, 2.42 ± 0.17 (*p* < 0.01) and 3.44 ± 0.28 Gy, respectively (Fig. 3f). Similarly, treatment with 1, 10 or 30 μM olaparib reduced IC_50_ values to 3.54 ± 0.14, 1.89 ± 0.09 (*p* < 0.001) and 2.09 ± 0.47 Gy (*p* < 0.01), respectively. These results suggest a plateau at 10 μM rucaparib or olaparib, with respect to clonogenic kill.

The dose enhancement factor (DEF_50_) was calculated as the radiation dose required to achieve 50 % kill in the absence of drug divided by the radiation dose required to kill cells in the presence of drug. Therefore, a DEF_50_ greater than 1 is indicative of radiosensitisation. Both PARP-1 inhibitors radiosensitised SK-N-BE(2c) and UVW/NAT cells. In the case of SK-N-BE(2c) cells, the DEF_50_ values were 2.01 and 1.22 following 10 μM rucaparib and olaparib treatment, respectively (Fig. 3c). In UVW/NAT cells, the corresponding values were 1.76 and 2.27 for 10 μM rucaparib and olaparib treatment, respectively (Fig. 3f). Dose enhancement was not further increased by treatment of cells with rucaparib or olaparib at concentrations greater than 10 μM.

**Fig. 4:**
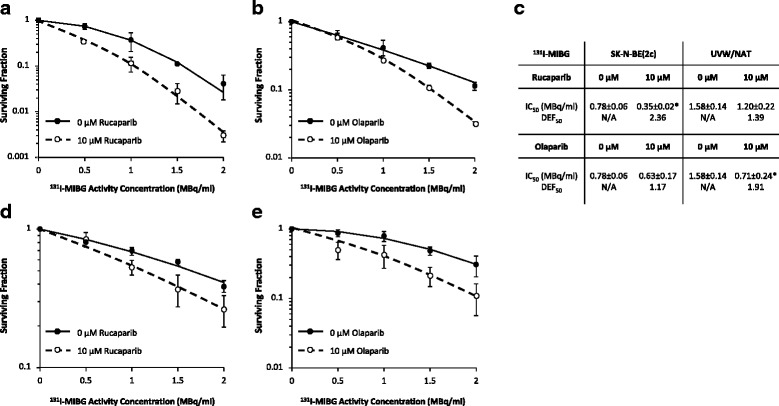
The effect of PARP-1 inhibition in combination with ^131^I-MIBG treatment. SK-N-BE(2c) (**a**, **b**) and UVW/NAT cells (**d**, **e**) were treated simultaneously with rucaparib (**a**, **d**) or olaparib (**b**, **e**) in combination with ^131^I-MIBG. Cells were treated with 10 μM rucaparib or olaparib following exposure to ^131^I-MIBG at various activity concentrations, for a total of 24 h prior to seeding cells for clonogenic assay. The activity concentrations associated with 50 % cell kill (IC_50_) and the dose enhancement factors corresponding to 50 % clonogenic kill (DEF_50_) are shown (**c**). Data are means ± SEM, *n* = 3; *p < 0.05 compared to the IC_50_ in the absence of drug

Rucaparib and olaparib also sensitised SK-N-BE(2c) and UVW/NAT cells to treatment with ^131^I-MIBG (Fig. 4). In SK-N-BE(2c) cells, the ^131^I-MIBG activity concentration corresponding to the IC_50_ was reduced from 0.78 ± 0.06 MBq/ml to 0.35 ± 0.02 (*p* < 0.05) or 0.63 ± 0.17 MBq/ml after treatment with 10 μM rucaparib or olaparib, respectively (Fig. 4c). Similarly, in UVW/NAT cells, the corresponding IC_50_ values were 1.58 ± 0.14 MBq/ml for ^131^I-MIBG treatment alone and 1.20 ± 0.22 and 0.71 ± 0.24 MBq (*p* < 0.05) in the presence of rucaparib or olaparib, respectively. DEF_50_ values were calculated as 2.36 and 1.17 in SK-N-BE(2c) cells treated with 10 μM rucaparib or olaparib respectively (Fig. 4c). In UVW/NAT cells, DEF_50_ values obtained were 1.39 and 1.91 following treatment with 10 μM rucaparib or olaparib, respectively.

### PARP-1 inhibition induces supra-additive cell kill in combination with X-irradiation or ^131^I-MIBG

**Fig. 5:**
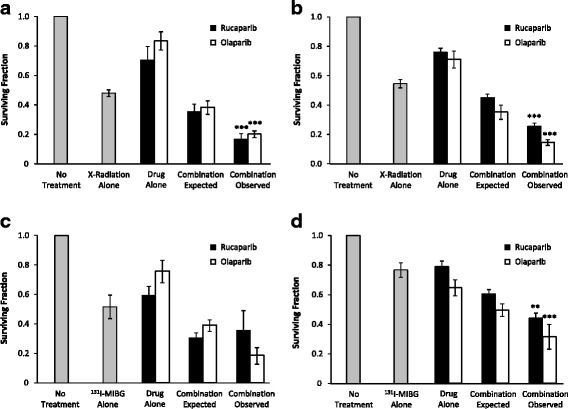
Clonogenic survival following the treatment of SK-N-BE(2c) and UVW/NAT cells with rucaparib or olaparib and X-radiation or ^131^I-MIBG as single agent modalities or in combination. SK-N-BE(2c) (**a**, **c**) and UVW/NAT cells (**b**, **d**) were treated with 10 μM rucaparib (black bars), 10 μM olaparib (white bars), 3 Gy X-radiation (**a**, **b**) or 1 MBq/ml ^131^I-MIBG (**c**, **d**), as single agents or in combination. The outcome of the latter treatment is designated as ‘combination observed’ in the figure. Cells were incubated for 24 h and survival was assessed by clonogenic assay. The expected surviving fraction, if the two treatments had an additive effect with respect to clonogenic cell kill, was calculated as the product of the surviving fractions resulting from single agent treatments. This is designated as ‘combination expected’ in the figure. Data are means ± SEM, *n* = 4; **p < 0.01, *** *p* < 0.001 compared to 3 Gy X-irradiation (**a**, **b**) or 1 MBq/ml ^131^I-MIBG treatment (**c**, **d**)

The interaction between radiation treatment and PARP-1 inhibitors was further determined using X-irradiation doses (3 Gy) and activity concentrations (1 MBq/ml) that were responsible for 50 % kill of SK-N-BE(2c) clonogens. Combination treatment included rucaparib or olaparib at 10 μM. The expected clonogenic cell surviving fraction, if the two treatments had an additive effect, was calculated as the product of the surviving fractions resulting from single agent treatments. This is designated as “combination expected” in Fig. 5. The “combination observed” was the experimental surviving fraction following combination treatment.

Combined treatments produced significantly greater cell kill than single modality treatments. This was indicated by combination expected surviving fractions of 0.36 ± 0.05 or 0.38 ± 0.05 following an additive interaction between rucaparib or olaparib with X-irradiation, respectively, in SK-N-BE(2c) cells (Fig. 5a). The surviving fraction of the observed combination of rucaparib (0.17 ± 0.04; *p* < 0.05) or olaparib (0.20 ± 0.02; *p* < 0.01) with X-irradiation in SK-N-BE(2c) cells was less than that of the expected combination, indicating supra-additivity. Similarly, in UVW/NAT cells, the surviving fraction of the observed combination of rucaparib (0.25 ± 0.02) or olaparib (0.14 ± 0.02) with X-radiation was less than that of the expected combination (rucaparib: 0.45 ± 0.02; olaparib: 0.35 ± 0.05) (Fig. 5b).

Supra-additive clonogenic cell kill also resulted from combination treatments comprising PARP-1 inhibition and ^131^I-MIBG (Fig. 5c and d). The surviving fraction resulting from combination treatments of UVW/NAT cells (rucaparib: 0.44 ± 0.03; olaparib: 0.32 ± 0.08) was less than of the expected combination (rucaparib: 0.61 ± 0.03; olaparib: 0.50 ± 0.04) (Fig. 5d). These results indicate that combination therapy produced greater cell kill than the administration of either treatment modality alone. Moreover, the observed surviving fraction following combination therapy was less than that expected from an additive interaction. No significant difference was observed in surviving fraction between the two PARP-1 inhibitors following single agent or combination therapy.

### PARP-1 inhibition in combination with X-irradiation promoted G_2_/M arrest

**Fig. 6:**
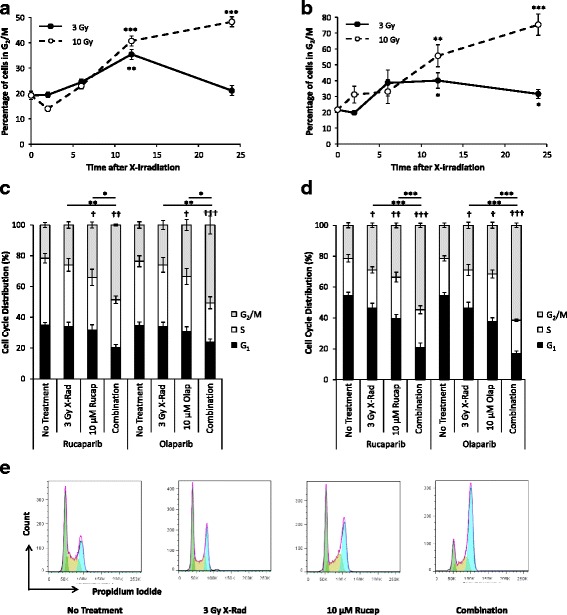
The effect of PARP-1 inhibition in combination with X-irradiation on cell cycle progression. SK-N-BE(2c) (**a**) and UVW/NAT (**b**) cells were irradiated with 3 or 10 Gy X-radiation before harvesting cells 2, 6, 12 and 24 h after irradiation. Cell cycle was analysed following by flow cytometry after staining with propidium iodide. Data are means ± SEM, *n* = 3; significance of differences: *p < 0.05, **p < 0.01, ***p < 0.001 compared to the unirradiated control at 0 h. SK-N-BE(2c) (**c**) and UVW/NAT (**d**) cells were treated with 10 μM rucaparib, 10 μM olaparib or 3 Gy X-radiation as single agents, or in combination. After 24 h, cells were fixed and cell cycle distribution was analysed by flow cytometry after staining with propidium iodide. Representative cell cycle profiles of UVW/NAT cells are shown in (**e**). Accumulation of cells in specific cell cycle phases was quantified using the Dean-Jet-Fox algorithm in FlowJo. G_1_, S and G_2_/M populations are highlighted in green, yellow and blue, respectively. Data are means ± SEM, *n* = 4; †*p* < 0.05, ††*p* < 0.01, †††*p* < 0.01 proportion of G_2_/M cells compared with untreated cells; *p < 0.05, **p < 0.01, ***p < 0.001 proportion of G_2_/M cells following combination treatment compared with each single agent treatment

Irradiation administered as a single agent promoted a significant increase in the G_2_/M cell population 12 h after irradiation, from 19 ± 1 % in SK-N-BE(2c) cells at 0 h, to 35 ± 1 % following 3 Gy irradiation (*p* < 0.01; Fig. 6a). However, 24 h after irradiation, the proportion of SK-N-BE(2c) cells in G_2_/M phase had decreased and was no longer significantly elevated relative to 0 h. Similarly, in UVW/NAT cells, the proportion of G_2_/M cells significantly increased from 22 ± 1 % at 0 h to 40 ± 5 % (*p* < 0.05) and 32 ± 3 % (*p* < 0.05), 12 h and 24 h after 3 Gy irradiation, respectively (Fig. 6b). In contrast, exposure to a radiation dose of 10 Gy caused a significant increase in the proportion of cells in G_2_/M phase at 12 h, which persisted at 24 h in both cell lines (Fig. 6a and b).

Rucaparib and olaparib single agent treatments significantly increased the G_2_/M population of SK-N-BE(2c) cells, from 24 ± 3 % in unirradiated controls, to 34 ± 3 % or 33 ± 3 % after administration of 10 μM rucaparib or olaparib, respectively (*p* < 0.05; Fig. 6c). Although radiation alone had no significant effect on cell cycle distribution 24 h after exposure, the effect of combination treatment was assessed at this time point to reflect the time at which combination treatments were assayed for clonogenic capacity. Combination treatment significantly increased the G_2_/M arrest observed with drug alone. This was indicated by an increase in the G_2_/M population to 49 ± 5 % (*p* < 0.001) and 51 ± 5 % (*p* < 0.001) following rucaparib or olaparib combination treatment, respectively, in SK-N-BE(2c) cells. Rucaparib and olaparib, both as single agents and as components of combination therapy, produced a similar level of G_2_/M arrest.

Similar treatment-induced, cell cycle redistribution was observed in UVW/NAT cells (Fig. 6d). Single agent rucaparib or olaparib treatment increased the G_2_/M population of UVW/NAT cells from 22 ± 1 % in unirradiated controls, to 32 ± 2 % (*p* < 0.01) or 30 ± 3 % (*p* < 0.05), respectively. Combination treatment significantly increased the G_2_/M population from 22 ± 1 % in unirradiated controls, to 55 ± 1 % (*p* < 0.001) and 61 ± 2 % (*p* < 0.001) following rucaparib or olaparib combination treatment, respectively. Representative histograms obtained using SK-N-BE(2c) cells are shown in Fig. 6e.

### PARP-1 inhibition prevented the restitution of radiation-induced DNA damage

**Fig. 7:**
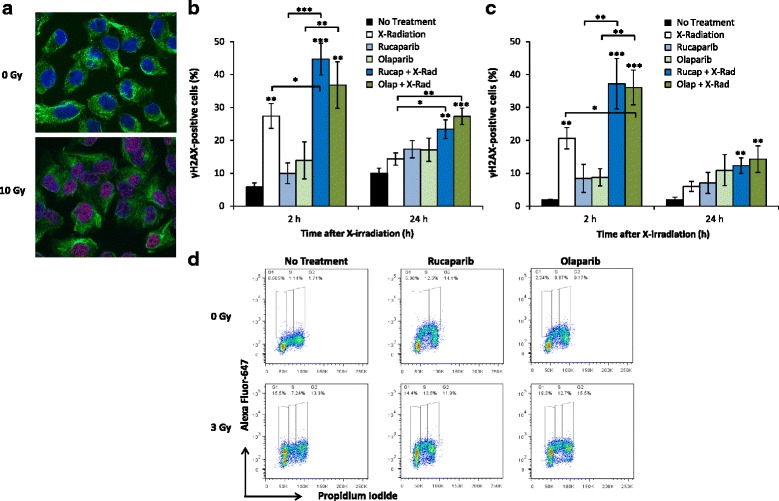
The effect of PARP-1 inhibition on the persistence of radiation-induced DNA damage. Immunofluorescent confocal microscopy confirmed nuclear localisation of anti-γH2AX Alexa-Fluor 647-labelled antibody (**a**) following 10 Gy X-radiation treatment (blue, DAPI nuclear stain; green, anti-β-tubulin cytoplasmic antibody visualised using Alexa Fluor 488-conjugated secondary antibody; red, anti-γH2AX Alexa Fluor 647-conjugated antibody). SK-N-BE(2c) (**b**) and UVW/NAT (**c**) cells were treated with 10 μM rucaparib, 10 μM olaparib or 3 Gy X-radiation as single agents, or in combination. Cells were harvested 2 and 24 h after irradiation and the total percentage of γH2AX positive cells was analysed using flow cytometry. Representative FACS dot plots obtained from SK-N-BE(2c) cells are shown (**d**). Data are means ± SEM, *n* = 3; *p < 0.05, **p < 0.01, ***p < 0.001 compared to untreated cells

The generation of γH2AX foci at the site of DNA double strand breaks follows phosphorylation of the H2AX histone variant protein at serine residue 139 [44]. γH2AX fluorescence intensity was proportional to the magnitude of DNA damage [45], and was detected with an anti-γH2AX Alexa Fluor 647-conjugated antibody. γH2AX foci co-localised with the DNA intercalating fluorescent stain DAPI, thereby confirming the nuclear location of γH2AX (Fig. 7a).

X-radiation treatment significantly increased DNA damage 2 h after irradiation (Fig. 7b, c and d). This was indicated by an increase from 6 to 27 % (*p* < 0.01) and from 2 to 21 % (*p* < 0.01) γH2AX positivity relative to total cellular DNA for SK-N-BE(2c) and UVW/NAT cells, respectively. Twenty four hours after irradiation, these values decreased to 14 % (SK-N-BE(2c)) and 6 % (UVW/NAT), indicating DNA repair. Combination treatment, consisting of rucaparib or olaparib with X-irradiation, resulted in greater DNA damage compared with irradiation alone. Combined X-irradiation with rucaparib caused an increase in γH2AX positivity to 45 % (*p* < 0.001) in SK-N-BE(2c) cells 2 h after treatment and, compared with irradiation alone, the damage was more sustained as indicated by 23 % γH2AX positivity (*p* < 0.05) 24 h after treatment. Similarly, combination treatment of cells with X-irradiation and olaparib caused 37 % DNA damage (*p* < 0.01) in SK-N-BE(2c) cells 2 h after treatment, which remained unrepaired (27 % DNA damage; *p* < 0.001) 24 h after treatment. Therefore, combining rucaparib or olaparib with X-irradiation produced a significantly greater amount of DNA damage, compared with X-irradiation alone (*p* < 0.05).

Similar inhibition of DNA damage repair was observed in UVW/NAT cells (Fig. 7c). Combining X-irradiation with rucaparib produced the greatest increase in γH2AX positivity compared with either single agent modality alone, exemplified by an increase in γH2AX from 2 and 3 % in untreated cells, to 37 % (*p* < 0.001) and 12 % (*p* < 0.01) 2 h and 24 h after treatment, respectively. Likewise, olaparib in combination with X-irradiation resulted in the greatest DNA damage 2 h after treatment (36 % γH2AX positivity; *p* < 0.01) compared with single agent treatments, which remained unrepaired 24 h after treatment (14 % γH2AX positivity; *p* < 0.001). Furthermore, combination treatment produced significantly greater initial DNA damage compared with X-irradiation alone (*p* < 0.05). These results indicate that PARP-1 inhibition prevented the restitution of radiation-induced DNA damage.

## Discussion

Patients with high-risk neuroblastoma have an overall survival rate of 40 % despite multi-modal treatment [2]. Therefore, they present a significant challenge to paediatric oncologists. Single agent treatment with ^131^I-MIBG is effective in the clinical management of high-risk neuroblastoma. However, recent studies indicate that maximal benefit will be achieved through its administration in combination with radiosensitising drugs [46–49]. In this study, we observed, in pre-clinical models of neuroblastoma, that the third generation PARP-1 inhibitors, rucaparib and olaparib, significantly enhanced the efficacy of ionising radiation, in the form of external beam X-rays or ^131^I-MIBG. Our results indicate that the mechanism of radiosensitisation entails prolonged DNA damage and accumulation of cells in G_2_/M phase of the cell cycle. PARP-1 inhibitors rucaparib and olaparib were comparable with respect to their potentiation of the lethality of X-irradiation or ^131^I-MIBG. Accordingly, both PARP-1 inhibitors may be considered of benefit to high-risk neuroblastoma patients undergoing targeted radiotherapy.

Since the discovery of the synthetic lethality of PARP-1 inhibition in cells deficient in homologous recombination (HR) [17, 18], there has been much interest in the therapeutic application of PARP-1 inhibitors. PARP-1 inhibitors have proven an effective monotherapy in *BRCA*-mutated breast cancer [31], ovarian cancer [32] and prostate cancer [33]. However, tumours proficient in HR repair may also be susceptible to treatment with PARP-1 inhibitors if administered in combination with cytotoxic drug therapy [34–36] and radiotherapy [50]. Here, we provide pre-clinical evidence supporting the use of PARP-1 inhibition in combination with external beam X-radiation or ^131^I-MIBG. The current study focused on rucaparib and olaparib, the first PARP-1 inhibitors to enter clinical trial [30–33, 36] and gain FDA approval, respectively.

Although the radiosensitising capacity of PARP-1 inhibitors has previously been demonstrated in vitro [22, 51–55], this is the first study to show synergism between rucaparib or olaparib with ^131^I-MIBG. Simultaneous treatment with 10 μM rucaparib or olaparib effectively halved the external beam X-radiation dose or the ^131^I-MIBG activity concentration required to kill 50 % of clonogens (IC_50_) derived from human neuroblastoma SK-N-BE(2c) cells, and human glioma UVW cells genetically engineered to express the noradrenaline transporter (NAT). Rucaparib or olaparib displayed similar radiosensitising potency. Furthermore, combination treatment produced greater than additive cell kill, indicating the potential for enhanced therapeutic benefit.

The present study demonstrated that rucaparib, olaparib and X-irradiation monotherapies significantly increased the proportion of cells in the G_2_/M phase of the cell cycle, which would also include a small proportion of cells in late S phase, and has been reported by others [56]. This is associated with increased radiosensitivity [57], due to the doubling of the amount of DNA susceptible to radiation trajectory following DNA synthesis in the preceding S phase. This indicates the importance of determining the optimal scheduling of the components of combination treatment to maximise therapeutic benefit. For example, we previously reported that simultaneous delivery of PJ34 (a second generation PARP-1 inhibitor), the topoisomerase inhibitor topotecan and ^131^I-MIBG maximised the efficacy of this 3-way combination [22]. Notably, olaparib-induced radiosensitisation was shown to be replication dependent [52], suggesting that the effects of PARP-1 inhibition would have greater effect in rapidly proliferating tumour cells [58].

The toxicity of PARP-1 inhibition is hypothesised to involve the accumulation of single strand breaks in irradiated cells, which are subsequently converted to double strand breaks upon collision with the advancing replication fork [52, 59]. Double strand breaks are quantified following analysis of γH2AX foci [45]. In response to genotoxic agents such as irradiation, the histone variant protein H2AX becomes phosphorylated at serine residue 139 at the site of double strand breaks [45]. We demonstrate here that, at cytotoxic concentrations, both PARP-1 inhibitors increased the accumulation of radiation-induced DNA damage and prevented the restitution of this damage 24 h after irradiation. Our results are supportive of others who also show that radiation-induced DNA damage remains unrepaired up to 22 h after irradiation following exposure to olaparib [55] or rucaparib [51]. Interestingly, Senra et al. hypothesised that olaparib-induced radiosensitisation was not only the result of impaired DNA repair, but also the result of olaparib-induced vasodilation [55]. The widening of tumour-associated blood vessels could be of therapeutic benefit, resulting in increased efficiency of drug delivery as well as well as re-oxygenation of hypoxic radioresistant regions of tumours [60, 61]. Significantly, rucaparib also causes vasodilation [60, 62].

The anti-tumour effect of PARP-1 inhibitors is not only due to inhibition of PARP-1 catalytic activity. PARP-1 inhibitor toxicity has also been attributed to ‘PARP trapping’, whereby PARP-1 is confined at the site of DNA damage, thus preventing DNA repair, replication and transcription, culminating in cell death [63]. Indeed, clinical stage PARP-1 inhibitors display a range of capacities to trap PARP-1 at the site of DNA damage, which could influence the selection of PARP-1 inhibitors for clinical use [64].

PARP-1 inhibitors are increasingly being considered for the treatment of neuroblastoma. Rucaparib has been shown to improve the efficacy of the alkylating agent temozolomide in neuroblastoma pre-clinical models [24]. The alternative PARP-1 inhibitor niraparib (formerly MK-4827) effectively sensitised a panel of neuroblastoma cells to external beam radiation. The degree of radiosensitisation was shown to be independent of *MYCN* amplification [65]. *MYCN* amplification occurs in 25 % of all primary neuroblastomas and is used for neuroblastoma risk stratification [2]. However, to our knowledge, this is the first study to demonstrate the radiosensitising potential of rucaparib and olaparib in combination with ^131^I-MIBG. Abnormalities in the non-homologous end joining repair pathway, such as increased PARP-1 and DNA Ligase protein expression, have been implicated in neuroblastoma cell survival and pathogenicity [37]. Indeed, increased PARP-1 expression was shown to correlate with increased genomic instability in neuroblastoma cell lines, including SK-N-BE(2c), and was also associated with higher neuroblastoma stage and poor overall survival [37], suggesting these tumours will be particularly susceptible to PARP-1 inhibition.

## Conclusions

We have demonstrated that the third generation PARP-1 inhibitors rucaparib and olaparib sensitised tumour cells to radiation treatment. This was manifest as a 50 % reduction in the X-radiation dose or ^131^I-MIBG activity concentration required to achieve 50 % cell kill. X-radiation-induced DNA damage was significantly increased 2 h after irradiation by combination with PARP-1 inhibitors. Moreover, combination treatment (i) prevented the restitution of DNA and (ii) induced greater G_2_/M cell cycle arrest than single agent modalities. Finally, rucaparib and olaparib were shown to be equipotent inhibitors of PARP-1 activity and displayed analogous levels of radiosensitisation in neuroblastoma models. Our findings suggest that the administration of PARP-1 inhibition and ^131^I-MIBG to high-risk neuroblastoma patients may be beneficial.
